# Logical Divergence, Logical Entropy, and Logical Mutual Information in Product MV-Algebras

**DOI:** 10.3390/e20020129

**Published:** 2018-02-16

**Authors:** Dagmar Markechová, Batool Mosapour, Abolfazl Ebrahimzadeh

**Affiliations:** 1 Department of Mathematics, Faculty of Natural Sciences, Constantine the Philosopher University in Nitra, A. Hlinku 1, SK-949 01 Nitra, Slovakia; 2 Department of Mathematics, Farhangian University, 7616914111 Kerman, Iran; 3 Young Researchers and Elite Club, Zahedan Branch, Islamic Azad University, 9816883673 Zahedan, Iran

**Keywords:** product MV-algebra, logical entropy, logical mutual information, logical cross entropy, logical divergence

## Abstract

In the paper we propose, using the logical entropy function, a new kind of entropy in product MV-algebras, namely the logical entropy and its conditional version. Fundamental characteristics of these quantities have been shown and subsequently, the results regarding the logical entropy have been used to define the logical mutual information of experiments in the studied case. In addition, we define the logical cross entropy and logical divergence for the examined situation and prove basic properties of the suggested quantities. To illustrate the results, we provide several numerical examples.

## 1. Introduction

In all areas of empirical research, it is very important to know how much information we gain by the realization of experiments. As it is known, the measure of information is entropy, the standard approach being based on Shannon entropy [1]. The standard mathematical model of an experiment in information theory [2] is a measurable partition of a probability space. Let us remind that a measurable partition of a probability space (X,S,P) is a sequence $A=\left\{A_{1},\ldots ,A_{n}\right\}$ of measurable subsets of X such that $\cup_{i=1}^{n}A_{i}=X$ and $A_{i}\cap A_{j}=\;\emptyset$ whenever i≠j. The Shannon entropy of the measurable partition $A=\left\{A_{1},\ldots ,A_{n}\right\}$ with probabilities $p_{i}=P(A_{i}),$ i=1,…,n, of the corresponding elements, is the number $h^{S}(A)=\sum_{i=1}^{n}S(p_{i}),$ where S:[0,1]→ℜ is the Shannon entropy function defined by the formula:

(1) $$S(x)=\;\left\{ \begin{array}{ll} -\;x\log x, & \mathrm{if}\;x>0;\\ 0, & \mathrm{if}\;x=0. \end{array}\right.$$

In classical theory, partitions are defined within the Cantor set theory. However, it has turned out that, in many cases, the partitions defined in the context of fuzzy set theory [3] are more suitable for solving real problems. Hence, numerous suggestions have been put forward to generalize the classical partitions to fuzzy partitions [4,5,6,7,8,9,10]. Fuzzy partitions provide a mathematical model of random experiments the outcomes of which are unclear, inaccurately defined events. The Shannon entropy of fuzzy partitions has been studied by many authors; we refer the reader to, e.g., [11,12,13,14,15,16,17,18,19,20,21].

The notion of an MV-algebra, originally proposed by Chang in [22] in order to give an algebraic counterpart of the Łukasiewicz many-valued logic [23] (MV = many valued), generalizes some classes of fuzzy sets. MV-algebras have been investigated by numerous international research groups [24,25,26,27,28]. A Shannon entropy theory for MV-algebras was created in [29,30]. The fuzzy set theory is a rapidly evolving field of theoretical and applied mathematical research. At present the subjects of intensive study are also other algebraic structures based on the fuzzy set theory, such as D-posets [31,32,33], effect algebras [34], and A-posets [35,36]. Some results concerning Shannon’s entropy on these structures have been provided, e.g., in [37,38,39].

An important case of MV-algebras is the so-called product MV-algebra (see, e.g., [40,41,42,43,44,45]). This notion was proposed independently by two authors: Riečan [40] and Montagna [41]. A Shannon entropy theory for product MV-algebras was provided in [30,46,47]. We note that in the recently published paper [48], the results regarding the Shannon entropy of partitions in product MV-algebras were exploited to define the notions of Kullback-Leibler divergence and mutual information of partitions in product MV-algebras. The Kullback-Leibler divergence (often shortened to K-L divergence) was proposed in [49] as the distance between two probability distributions and it is currently one of the most basic quantities in information theory.

When addressing some special issues instead of Shannon entropy, it is preferable to use an approach based on the conception of logical entropy [50,51,52,53,54,55,56]. If $A=\left\{A_{1},\ldots ,A_{n}\right\}$ is a measurable partition with probabilities $p_{1},\ldots ,p_{n}$ of the corresponding elements, then the logical entropy of A is defined by the formula $h^{l}(A)=\sum_{i=1}^{n}l(p_{i}),\;$ where l:[0,1]→[0,1] is the logical entropy function defined by:

(2) l(x)=x(1−x).

In [50], the author gives a history of the logical entropy formula $h^{l}(A)=1-\sum_{i=1}^{n}p_{i}^{2}.$ It is interesting that Alan Turing, who worked during the Second World War at the Bletchley Park facility in England, used the formula $\sum_{i=1}^{n}p_{i}^{2}$ in his famous cryptanalysis work. This formula was independently used by Polish crypto-analysts in their work [57] on the Enigma. The relationship between the Shannon entropy and the logical entropy is examined in [50]. In addition, the notions of logical cross entropy and logical divergence have been proposed in the cited paper. For some recent works related to the concept of logical entropy on algebraic structures based on fuzzy set theory, we refer the reader to (for example) [58,59,60,61,62,63,64,65].

The purpose of this article is to extend the study of logical entropy provided in [50] to the case of product MV-algebras. The remainder of the article is structured as follows. In Section 2 we present basic concepts, terminology and the known results that are used in the article. The results of the paper are given in the succeeding three sections. In Section 3, we define the logical entropy of partitions in product MV-algebras and its conditional version and examine their properties. In the following section, the results of Section 3 are exploited to define the concept of logical mutual information for the studied situation. Using the notion of logical conditional mutual information, we present chain rules for logical mutual information in product MV-algebras. In Section 5, we define the logical cross entropy and the logical divergence of states defined on product MV-algebras and we examine properties of these quantities. The results are explained with several examples to illustrate the theory developed in the article. The final section contains a brief overview. It is shown that by replacing the Shannon entropy function (Equation (1)) by the logical entropy function (Equation (2)) we obtain the results analogous to the results given in [48].

## 2. Preliminaries

The aim of the section is to provide basic concepts, terminology and the known results used in the paper.

**Definition** **1** [25]. *An MV-algebra is an algebraic structure* M=(M,⊕,⊗,⊥,0,1), *where* ⊕ *is a commutative and associative binary operation on* M,  ⊗ *is a binary operation on* M, ⊥ *is a unary operation on* M, 0,1∈M, *such that* a⊕0=a; a⊕1=1; ${\left(a^{⊥}\right)}^{⊥}=a;$ $0^{⊥}=1;$ $a⊕a^{⊥}=1;$ ${\left(a^{⊥}⊕b\right)}^{⊥}⊕b$ $={\left(a⊕b^{⊥}\right)}^{⊥}⊕a;$ a⊗b= ${\left(a^{⊥}⊕b^{⊥}\right)}^{⊥}.$

**Example** **1.** *Let* M *be the unit real interval* [0, 1], $a^{⊥}=1-a,$ a⊕b=min(a+b,1), a⊗b= max(a+b−1,0). *Then the system* (M,⊕,⊗,⊥ ,0,1) *is an MV-algebra*.

**Example** **2.** *Let* (L,+,≤) *be a commutative lattice ordered group (shortly l-group), i.e.,* (L,+) *is a commutative group,* (L,≤) *is a partially ordered set being a lattice and* a≤b ⟹ a+c≤b+c. *Let* u∈L *be a strong unit of* L *(i.e., to each* a∈L *there exists a positive integer* n *satisfying the condition* a≤nu) *such that* u>0, *where* 0 *is a neutral element of* (L,+). *Put* [0,u]={a∈L;  0≤a≤u}, $a^{⊥}=u-a,$ a⊕b=(a+b)∧u, a⊗b= (a+b−u)∨0, 1=u. *Then the system* $M_{0}(L,u)=([0,u],⊕,⊗,⊥,0,1)$ *is an MV-algebra. Evidently, if* a,b∈L *such that* a+b≤u, *then* a⊕b=a+b *Moreover, it can be seen that the condition* a⊗b=0 *is equivalent to the condition that* a+b≤u.

By the following Mundici representation theorem, every MV-algebra M can be identified with the unit interval [0, *u*] of a unique (up to isomorphism) commutative lattice ordered group L with a strong unit *u*. We say that L is the *l*-group corresponding to M.

**Theorem** **1** [66]. *Let* M *be an MV-algebra. Then there exists a commutative lattice ordered group* L *with a strong unit u such that* $M=M_{0}(L,u),$ *and* (*L*, *u*) *is unique up to isomorphism.*

**Definition** **2** [47]. *Let* M=(M,⊕,⊗,⊥,0,1) *be an MV-algebra. A partition in M is an n-tuple* $\alpha =(a_{1},\ldots ,a_{n})$ *of elements of* M *with the property* $a_{1}+\ldots +a_{n}=u,$ *where* + *is an addition in the l-group* L *corresponding to* M *and* u *is a strong unit of* L.

In the paper we shall deal with product MV-algebras. The definition of product MV-algebra (cf. [40,41]), as well as the previous definition of partition in MV-algebra, is based on Mundici’s theorem, i.e., the MV-algebra operation ⊕ in the following definition, and in what follows, is substituted by the group operation + in the commutative lattice ordered group L that corresponds to the considered MV-algebra M. Analogously, the element *u* is a strong unit of L and ≤ is the partial-ordering relation in L.

**Definition** **3** [40]. *A product MV-algebra is an algebraic structure* (M,⊕,⊗,·,⊥,0,1) *where* (M,⊕,⊗,⊥,0,1) *is an MV-algebra and* ⋅ *is a commutative and associative binary operation on* M *with the following properties:*

(*i*) *for every* a∈M, u⋅a=a;
(*ii*) *if* a,b,c∈M *such that* a+b≤u, *then* c⋅a+c⋅b≤u, *and* c⋅(a+b)=c⋅a+c⋅b.

For brevity, we will write (M,⋅ ) instead of (M,⊕,⊗,·,⊥,0,1) Further, we consider a state defined on (M,⋅ ) which plays the role of a probability measure on *M*. We note that a relevant probability theory for the product MV-algebras was developed in [44], see also [27,45].

**Definition** **4** [44]. *A state on a product MV-algebra* (M,⋅ ) *is a map* s:M→[0,1] *with the properties*:

(*i*) s(u)=1;
(*ii*) *if* a,b∈M *such that* a+b≤u, *then* s(a+b)=s(a)+s(b).

Notice that the disjointness of the elements a,b∈M is expressed in the previous definition by the condition a+b≤u (or equivalently by a≤u−b). According to the Mundici theorem this condition can be formulated in the equivalent way as a+b∈M or also as a⊗b=0. As is customary, we will write $\sum_{i=1}^{n}a_{i}$ instead of $a_{1}+\ldots +a_{n}.$ Let s:M→[0,1] be a state. Applying induction we get that for any elements $a_{1},\ldots ,a_{n}\in M$ such that $\sum_{i=1}^{n}a_{i}\leq u,$ it holds $s\left(\sum_{i=1}^{n}a_{i}\right)=\sum_{i=1}^{n}s(a_{i}).$

In the system of all partitions of (M,⋅ ), we define the refinement partial order ≻ in a standard way (cf. [23]). If $\alpha =(a_{1},\ldots ,a_{n}),$ and $\beta =(b_{1},\ldots ,b_{m})$ are two partitions of (M,⋅ ), then we write β≻α (and we say that β is a refinement of α), if there exists a partition {I(1),I(2),…,I(n)} of the set {1,2,…,m} such that $a_{i}=\sum_{j\in I\left(i\right)}b_{j},$ for i=1,…,n. Further, we define α∨β= $(a_{i}\cdot b_{j};\;i=1,\ldots ,n,j=1,2,\ldots ,m).$ Since $\sum_{i=1}^{n}\sum_{j=1}^{m}a_{i}\cdot b_{j}$ $=\left(\sum_{i=1}^{n}a_{i}\right)\cdot \left(\sum_{j=1}^{m}b_{j}\right)$ =u⋅u=u, the system α∨β is a partition of (M,⋅ ). The partition α∨β represents a combined experiment consisting of a realization of the considered experiments α and β. If $\alpha_{1},\alpha_{2},\ldots ,\alpha_{n}$ are partitions in a product MV-algebra (M,⋅ ), then we put $\vee_{i=1}^{n}\alpha_{i}=\alpha_{1}\vee \alpha_{2}\vee \ldots \vee \alpha_{n}.$

## 3. Logical Entropy of Partitions in Product MV-Algebras

In this section we define the logical entropy and the logical conditional entropy of partitions in a product MV-algebra and derive their properties.

**Definition** **5.** *Let* $\alpha =(a_{1},\ldots ,a_{n})$ *be a partition in a product MV-algebra* (M,⋅ ), *and* s:M→[0,1] *be a state. Then we define the logical entropy of* α *with respect to state s by the formula:*

(3) $$h_{s}^{l}(\alpha )=\sum_{i=1}^{n}s(a_{i})\;(1-s(a_{i})).\;$$

**Remark** **1.** *Evidently, the logical entropy* $h_{s}^{l}(\alpha )$ *is always nonnegative, and it has the maximum value* $1-\frac{1}{n}$ *for the state* s *uniform over* $\alpha =(a_{1},\ldots ,a_{n}).$ *Since* $\sum_{i=1}^{n}s(a_{i})\;=s\left(\sum_{i=1}^{n}a_{i}\right)=s(u)=1,$ *Equation (3) can also be written in the following form:*

(4) $$h_{s}^{l}(\alpha )=1-\sum_{i=1}^{n}{\left(s(a_{i})\right)}^{2}.$$

**Example** **3.** *Let* (M,⋅ ) *be a product MV-algebra and* s:M→[0,1] *be a state. If we put* ε=(u), *then* ε *is a partition of* (M,⋅ ) *with the property* α≻ε, *for every partition* α *of* (M,⋅ ). *Its logical entropy is* $h_{s}^{l}(\varepsilon )=0.$ *Let* a∈M *with* s(a)=p, *where* p∈(0,1). *It is obvious that the pair* α=(a,u−a) *is a partition of* (M,⋅ ). *Since* s(u−a)=1−p, *the logical entropy* $h_{s}^{l}(\alpha )=2p(1-p).$ *If we put* $p=\frac{1}{2},$ *then we have* $h_{s}^{l}(\alpha )=\frac{1}{2}.$

In the proofs we shall use the following propositions.

**Proposition** **1.** *Let* $\alpha =(a_{1},\ldots ,a_{n})$ *be a partition of* (M,⋅ ). *Then, for every* b∈M, *we have:*

$$s(b)=\sum_{i=1}^{n}s(a_{i}\cdot b).$$

**Proof.**  According to Definitions 2, 3, and 4 we obtain:

$$s(b)=s(u\cdot b)=s((\sum_{i=1}^{n}a_{i})\cdot b)=s(\sum_{i=1}^{n}a_{i}\cdot b)=\sum_{i=1}^{n}s(a_{i}\cdot b).$$

 ☐

**Proposition** **2.** *For arbitrary partitions* α,β *of* (M,⋅ ), *it holds* α∨β≻α.

**Proof.**  Let us suppose that $\alpha =(a_{1},\ldots ,a_{n}),$ $\beta =(b_{1},\ldots ,b_{m}).$ Put I(i)={(i,1),…,(i,m)}, for i=1,…,n. Since we have:

$$a_{i}=a_{i}\cdot u=a_{i}\cdot \left(\sum_{j=1}^{m}b_{j}\right)\;=\sum_{j=1}^{m}a_{i}\cdot b_{j}=\sum_{\left(l,j)\in I(i\right)}a_{l}\cdot b_{j},$$

for i=1,…,n, we conclude that α∨β≻α. ☐

**Proposition** **3.** *Let* α,β *be partitions of* (M,⋅ ) *such that* β≻α. *Then for an arbitrary partition* γ, *it holds* β∨γ≻α∨γ.

**Proof.**  Let $\alpha =(a_{1},\ldots ,a_{n}),$ $\beta =(b_{1},\ldots ,b_{m}),$ $\gamma =(c_{1},\ldots ,c_{r}),$ β≻α. Then there is a partition {I(1),I(2),…,I(n)} of the set {1,2,…,m} such that $a_{i}=\sum_{j\in I\left(i\right)}b_{j},$ for i=1,…,n. A partition α∨γ $=\left(a_{i}\cdot c_{k};\;i=1,\ldots ,n,k=1,\ldots ,r\right)$ is indexed by {(i, k);  i=1,…,n,k=1,…,r}, therefore we put I(i,k)= {(j, k);  j∈I(i)}, for i=1,…,n, k=1,…,r. We obtain:

$$a_{i}\cdot c_{k}=\left(\sum_{j\in I(i)}b_{j}\right)\cdot c_{k}=\sum_{j\in I(i)}b_{j}\cdot c_{k}=\sum_{\left(j,\;l)\in I(i,\;k\right)}b_{j}\cdot c_{l},$$

for i=1,…,n, k=1,…,r. This implies that β∨γ≻α∨γ. ☐

**Definition** **6.** *If* $\alpha =(a_{1},\ldots ,a_{n})$ *and* $\beta =(b_{1},\ldots ,b_{m})$ *are partitions of* (M,⋅ ), *then the logical conditional entropy of* α *given* β *is defined by:*

(5) $$h_{s}^{l}(\alpha /\beta )=\sum_{i=1}^{n}\sum_{j=1}^{m}s(a_{i}\cdot b_{j})\cdot (s(b_{j})-s(a_{i}\cdot b_{j})).$$

**Remark** **2.** *Since by Proposition 1, for* j=1,…,m, *it holds* $\sum_{i=1}^{n}s(a_{i}\cdot b_{j})=\;s(b_{j}),$ *Equation (5) can be written in the following equivalent form:*

(6) $$h_{s}^{l}(\alpha /\beta )=\sum_{j=1}^{m}{\left(s(b_{j})\right)}^{2}-\sum_{i=1}^{n}\sum_{j=1}^{m}{\left(s(a_{i}\cdot b_{j})\right)}^{2}.$$

**Remark** **3.** *Since* $s(a_{i}\cdot b_{j})\leq s(b_{j}),$ *for* i=1,…,n,j=1,…,m, *the logical conditional entropy* $h_{s}^{l}(\alpha /\beta )$ *is always nonnegative. Let us consider the partition* ε=(u). *It can be easily verified that* $h_{s}^{l}(\alpha /\varepsilon )=h_{s}^{l}(\alpha ).$

**Theorem** **2.** *For arbitrary partitions* α,β *of* (M,⋅ ), *it holds:*

(7) $$h_{s}^{l}(\alpha \vee \beta )=h_{s}^{l}(\alpha )+h_{s}^{l}(\beta /\alpha ).$$

**Proof.**  Let us suppose that $\alpha =(a_{1},\ldots ,a_{n}),$ $\beta =(b_{1},\ldots ,b_{m}).$ Then by Equations (4) and (6) we obtain:

$$\begin{array}{c} h_{s}^{l}(\alpha )+h_{s}^{l}(\beta /\alpha )=1-\sum_{i=1}^{n}{\left(s(a_{i})\right)}^{2}+\;\sum_{i=1}^{n}\;{\left(s(a_{i})\right)}^{2}\;-\;\sum_{i=1}^{n}\sum_{j=1}^{m}{\left(s(a_{i}\cdot b_{j})\right)}^{2}\\ =1-\sum_{i=1}^{n}\sum_{j=1}^{m}{\left(s(a_{i}\cdot b_{j})\right)}^{2}=h_{s}^{l}(\alpha \vee \beta ). \end{array}$$

 ☐

**Remark** **4.** *Let* $\alpha_{1},\alpha_{2},\ldots ,\alpha_{n}$ *be partitions of* (M,⋅ ). *Using now Equation (7), considering the partition* $\vee_{i=1}^{n}\alpha_{i}=\alpha_{1}\vee \alpha_{2}\vee \ldots \vee \alpha_{n}$ *and applying induction, we get:*

(8) $$h_{s}^{l}(\alpha_{1}\vee \ldots \vee \alpha_{n})=h_{s}^{l}(\alpha_{1})+\sum_{i=2}^{n}h_{s}^{l}(\alpha_{i}/\alpha_{1}\vee \ldots \vee \alpha_{i-1}).$$

**Theorem** **3.** *For arbitrary partitions* α,β *of* (M,⋅ ), *it holds:*

(*i*) $h_{s}^{l}(\alpha /\beta )\leq h_{s}^{l}(\alpha );$
(*ii*) $h_{s}^{l}(\alpha \vee \beta )\leq h_{s}^{l}(\alpha )+h_{s}^{l}(\beta ).$

**Proof.**  Let us suppose that $\alpha =(a_{1},\ldots ,a_{n}),$ $\beta =(b_{1},\ldots ,b_{m}).$

(i) Since by Proposition 1, for i=1,…,n, it holds $\sum_{j=1}^{m}s(a_{i}\cdot b_{j})=s(a_{i}),$ we obtain:
  $$\sum_{j=1}^{m}s(a_{i}\cdot b_{j})\cdot (s(b_{j})-s(a_{i}\cdot b_{j}))\leq \left(\sum_{j=1}^{m}s(a_{i}\cdot b_{j})\right)\;\left(\sum_{j=1}^{m}\left(s(b_{j})-s(a_{i}\cdot b_{j})\right)\right)=s(a_{i})\;\left(\sum_{j=1}^{m}s(b_{j})-\sum_{j=1}^{m}s(a_{i}\cdot b_{j})\right)=s(a_{i})(1-s(a_{i})).$$
  Therefore:
  $$h_{s}^{l}(\alpha /\beta )=\sum_{i=1}^{n}\sum_{j=1}^{m}s(a_{i}\cdot b_{j})\cdot (s(b_{j})-s(a_{i}\cdot b_{j}))\leq \;\sum_{i=1}^{n}s(a_{i})(1-s(a_{i}))=h_{s}^{l}(\alpha ).\;$$
(ii) Combining Equation (7) and the previous property we obtain the claim (ii). ☐

In the following example, we illustrate the results of Theorem 3.

**Example** **4.** *Let us consider the measurable space* ([0, 1],B), *where* B *is the* σ− *algebra of all Borel subsets of the unit interval* [0,1]. *Put* $M=\left\{I_{A};\;A\in B\right\},$ *where* $I_{A}$ *is the indicator of the set* A, *and define, for every* $I_{A},I_{B}\in M,$ *the operation* ⋅ *by the equality* $I_{A}\cdot \;I_{B}=I_{A\cap B}.$ *The system* (M,⋅ ) *is a product MV-algebra with the unit element* $u=I_{X}.$ *Let us define a state* s:  M→[0,1] *by the equality* $s(I_{A})=$ $\int_{0}^{1}I_{A}(x)dx,$ *for any element* $I_{A}$ *of* M. *The pairs* $\alpha =\left(I_{\left[0,\;\frac{1}{2}\right]},\;I_{\left[\frac{1}{2},\;1\right]}\right)$ *and* $\beta =\left(I_{\left[0,\;\frac{1}{3}\right]},\;I_{\left[\frac{1}{3},\;1\right]}\right)$ *are partitions of* (M,⋅ ) *with the s-state values* $\frac{1}{2},\frac{1}{2}$ *and* $\frac{1}{3},\frac{2}{3}$ *of the corresponding elements, respectively. By Equation* (4) *we can easily calculate their logical entropy:* $h_{s}^{l}(\alpha )=\frac{1}{2},$ $h_{s}^{l}(\beta )=\frac{4}{9}.$ *The partition* α∨β= $\left(I_{\left[0,\;\frac{1}{3}\right]},I_{\left[\frac{1}{3},\;\frac{1}{2}\right]},\;I_{\left[\frac{1}{2},\;1\right]},\;0\right)$ *has the s-state values* $\frac{1}{3},\frac{1}{6},\frac{1}{2},0$ *of the corresponding elements, and the logical entropy:*

$$h_{s}^{l}(\alpha \vee \beta )=1-\left[{\left(\frac{1}{3}\right)}^{2}+{\left(\frac{1}{6}\right)}^{2}+{\left(\frac{1}{2}\right)}^{2}\right]=\frac{11}{18}.$$

*Since* $\frac{11}{18}\leq \frac{1}{2}+\frac{4}{9}$, *the inequality* $h_{s}^{l}(\alpha \vee \beta )\leq h_{s}^{l}(\alpha )+h_{s}^{l}(\beta )$ *holds. The logical conditional entropy of* α *given* β *is the number:*

$$h_{s}^{l}(\alpha /\beta )=\sum_{j=1}^{2}{\left(s(b_{j})\right)}^{2}-\sum_{i=1}^{2}\sum_{j=1}^{2}{\left(s(a_{i}\cdot b_{j})\right)}^{2}={\left(\frac{1}{3}\right)}^{2}+{\left(\frac{2}{3}\right)}^{2}-\left[{\left(\frac{1}{3}\right)}^{2}+{\left(\frac{1}{6}\right)}^{2}+{\left(\frac{1}{2}\right)}^{2}\right]=\frac{5}{9}-\frac{7}{18}=\frac{1}{6};$$

*analogously we get the logical conditional entropy* $h_{s}^{l}(\beta /\alpha )=\frac{1}{2}-\frac{7}{18}=\frac{1}{9}.$ *It can be verified that:*

$$h_{s}^{l}(\alpha \vee \beta )=h_{s}^{l}(\beta )+h_{s}^{l}(\alpha /\beta )=h_{s}^{l}(\alpha )+h_{s}^{l}(\beta /\alpha ).$$

**Theorem** **4.** *For arbitrary partitions* α,β,γ *of* (M,⋅ ), *it holds:*

(9) $$h_{s}^{l}(\alpha \vee \beta /\gamma )=h_{s}^{l}(\alpha /\gamma )+h_{s}^{l}(\beta /\alpha \vee \gamma ).$$

**Proof.**  Let us suppose that $\alpha =(a_{1},\ldots ,a_{p}),$ $\beta =(b_{1},\ldots ,b_{q}),$ $\gamma =(c_{1},\ldots ,c_{r}).$ Using Equation (6) we can write:

$$\begin{array}{ll} h_{s}^{l}(\alpha /\gamma )+h_{s}^{l}(\beta /\alpha \vee \gamma ) & =\sum_{k=1}^{r}{\left(s(c_{k})\right)}^{2}-\sum_{i=1}^{p}\sum_{k=1}^{r}{\left(s(a_{i}\cdot c_{k})\right)}^{2}+\sum_{i=1}^{p}\sum_{k=1}^{r}{\left(s(a_{i}\cdot c_{k})\right)}^{2}-\sum_{j=1}^{q}\sum_{i=1}^{p}\sum_{k=1}^{r}{\left(s(b_{j}\cdot a_{i}\cdot c_{k})\right)}^{2}\\ & =\sum_{k=1}^{r}{\left(s(c_{k})\right)}^{2}-\sum_{i=1}^{p}\sum_{j=1}^{q}\sum_{k=1}^{r}\left(s(a_{i}\cdot b_{j}\cdot c_{k}\right){)}^{2}=h_{s}^{l}(\alpha \vee \beta /\gamma ). \end{array}$$

 ☐

**Remark** **5.** *Let* $\alpha_{1},\alpha_{2},\ldots ,\alpha_{n},\gamma$ *be partitions of* (M,⋅ ). *Using the principle of mathematical induction, we get the following generalization of Equation (9):*

(10) $$h_{s}^{l}(\alpha_{1}\vee \ldots \vee \alpha_{n}/\gamma )=h_{s}^{l}(\alpha_{1}/\gamma )+\sum_{i=2}^{n}h_{s}^{l}(\alpha_{i}/\alpha_{1}\vee \ldots \vee \alpha_{i-1}\vee \gamma ).$$

*If we put* γ=ε, *as a special case of Equation (10) we get Equation (8).*

**Theorem** **5.** *For arbitrary partitions* α,β,γ *of* (M,⋅ ), *it holds:*

(*i*) β≻α *implies* $h_{s}^{l}(\beta )\geq h_{s}^{l}(\alpha );$
(*ii*) $h_{s}^{l}(\alpha \vee \beta )\geq \max\;[h_{s}^{l}(\alpha );\;h_{s}^{l}(\beta )];$
(*iii*) β≻α *implies* $h_{s}^{l}(\beta /\gamma )\geq h_{s}^{l}(\alpha /\gamma ).$

**Proof.** 

(i) Let $\alpha =(a_{1},\ldots ,a_{k}),$ $\beta =(b_{1},\ldots ,b_{l}).$ By the assumption that β≻α there is a partition {I(1),I(2),…,I(k)} of the set {1,2,…,l} with the property $a_{i}=\sum_{j\in I\left(i\right)}b_{j},$ for i=1,…,k. Therefore:
  $$\begin{array}{c} h_{s}^{l}(\alpha )=1-\sum_{i=1}^{k}{\left(s(a_{i})\right)}^{2}=1-\sum_{i=1}^{k}{\left(s(\sum_{j\in I\left(i\right)}b_{j})\right)}^{2}=1-\sum_{i=1}^{k}{\left(\sum_{j\in I(i)}s(b_{j})\right)}^{2}\\ \leq 1-\sum_{i=1}^{k}\sum_{j\in I(i)}{\left(s(b_{j})\right)}^{2}=1-\sum_{j=1}^{l}{\left(s(b_{j})\right)}^{2}=h_{s}^{l}(\beta ). \end{array}$$
  We used the inequality ${\left(\sum_{j\in I(i)}s(b_{j})\right)}^{2}\geq \sum_{j\in I(i)}{\left(s(b_{j})\right)}^{2}$ that follows from the inequality ${\left(x_{1}+\ldots +x_{n}\right)}^{2}\geq x_{1}^{2}+\ldots +x_{n}^{2}$ applicable for all nonnegative real numbers $x_{1},\ldots ,x_{n}.$
(ii) According to Proposition 2 it holds α∨β≻α, and α∨β≻β, therefore, the property (ii) is a direct consequence of the property (i).
(iii) Let β≻α. Then by Proposition 3 we have β∨γ≻α∨γ. Therefore, using Equation (7) and the property (i) we get:
  $$h_{s}^{l}(\beta /\gamma )=h_{s}^{l}(\beta \vee \gamma )-h_{s}^{l}(\gamma )\geq h_{s}^{l}(\alpha \vee \gamma )-h_{s}^{l}(\gamma )=h_{s}^{l}(\alpha /\gamma ).$$

 ☐

In the following theorem, we prove the concavity of logical entropy $h_{s}^{l}(\alpha )$ as a function of *s*. By the symbol S(M) we will denote the family of all states defined on *M*. It is easy to verify that if $s_{1},s_{2}\in S(M),$ then, for every real number λ∈[0,1], it holds that $\lambda \;s_{1}+(1-\lambda )\;s_{2}\in S(M).$

**Theorem** **6.** *Let* α *be a given partition of* (M,⋅ ). *Then, for every* $s_{1},s_{2}\in S(M)$, *and for every real number* λ∈[0,1], *it holds*:

$$\lambda \;h_{s_{1}}^{l}(\alpha )+\left(1-\lambda \right)\;h_{s_{2}}^{l}(\alpha )\leq h_{\lambda \;s_{1}+(1-\lambda )s_{2}}^{l}\;(\alpha ).$$

**Proof.**  Let $\alpha =(a_{1},\ldots ,a_{n}).$ The function ϕ:  ℜ→ℜ defined by $\phi (x)=x^{2},$ for every x∈ℜ, is convex, therefore, for every real number λ∈[0,1] and i=1,2,…,n, we have:

$${\left(\lambda \;s_{1}(a_{i})+(1-\lambda )\;s_{2}(a_{i})\right)}^{2}\leq \lambda {\left(\;s_{1}(a_{i})\right)}^{2}+(1-\lambda )\;{\left(s_{2}(a_{i})\right)}^{2}.$$

Hence, we obtain:

$$\sum_{i=1}^{n}{\left(\lambda \;s_{1}(a_{i})+(1-\lambda )\;s_{2}(a_{i})\right)}^{2}\leq \lambda \sum_{i=1}^{n}{\left(\;s_{1}(a_{i})\right)}^{2}+(1-\lambda )\;\sum_{i=1}^{n}{\left(s_{2}(a_{i})\right)}^{2},$$

and, consequently:

$$1-\sum_{i=1}^{n}{\left(\lambda \;s_{1}(a_{i})+(1-\lambda )\;s_{2}(a_{i})\right)}^{2}\geq 1-\lambda \sum_{i=1}^{n}{\left(\;s_{1}(a_{i})\right)}^{2}-(1-\lambda )\;\sum_{i=1}^{n}{\left(s_{2}(a_{i})\right)}^{2}.$$

Therefore, we can write:

$$\begin{array}{c} \lambda \;h_{s_{1}}^{l}(\alpha )+\left(1-\lambda \right)\;h_{s_{2}}^{l}(\alpha )=\lambda \left[1-\sum_{i=1}^{n}{\left(\;s_{1}(a_{i})\right)}^{2}\right]+(1-\lambda )\;\left[1-\sum_{i=1}^{n}{\left(s_{2}(a_{i})\right)}^{2}\right]\\ =1-\lambda \sum_{i=1}^{n}{\left(\;s_{1}(a_{i})\right)}^{2}-(1-\lambda )\;\sum_{i=1}^{n}{\left(s_{2}(a_{i})\right)}^{2}\\ \leq 1-\sum_{i=1}^{n}{\left(\lambda \;s_{1}(a_{i})+(1-\lambda )\;s_{2}(a_{i})\right)}^{2}\\ =1-\sum_{i=1}^{n}{\left(\lambda \;s_{1}+(1-\lambda )\;s_{2})(a_{i})\right)}^{2}=h_{\lambda \;s_{1}+(1-\lambda )s_{2}}^{l}\;(\alpha ). \end{array}$$

The result proves that the logical entropy $s\mapsto h_{s}^{l}\left(\alpha \right)$ is concave on the class S(M). ☐

## 4. Logical Mutual Information in Product MV-Algebras

In this section, the previous results are exploited to introduce the concept of logical mutual information of partitions in product MV-algebras and its conditional version and to derive their properties. In particular, using the concept of logical conditional mutual information we formulate chain rules for the examined situation.

**Definition** **7.** *Let* (M,⋅ ) *be a product MV-algebra. The logical mutual information of partitions* α *and* β *in* (M,⋅ ) *is defined by:*

(11) $$I_{s}^{l}(\alpha ,\beta )=h_{s}^{l}(\alpha )-h_{s}^{l}(\alpha /\beta ).$$

**Remark** **6.** *The inequality* $h_{s}^{l}(\alpha /\beta )\leq h_{s}^{l}(\alpha )$ *implies that the logical mutual information* $I_{s}^{l}(\alpha ,\beta )$ *is always nonnegative. Since, by Equation (7), it holds* $h_{s}^{l}(\alpha /\beta )=h_{s}^{l}(\alpha \vee \beta )-h_{s}^{l}(\beta ),$ *we also have the following identity:*

(12) $$I_{s}^{l}(\alpha ,\beta )=h_{s}^{l}(\alpha )+h_{s}^{l}(\beta )-h_{s}^{l}(\alpha \vee \beta ).$$

*Thereafter we can see that* $I_{s}^{l}(\alpha ,\beta )=I_{s}^{l}(\beta ,\alpha ),$ *and, due to the inequality* $h_{s}^{l}(\alpha )\leq h_{s}^{l}(\alpha \vee \beta )$ *(Theorem 5, (ii)), we have* $I_{s}^{l}(\alpha ,\beta )\leq \min[h_{s}^{l}(\alpha );\;h_{s}^{l}(\beta )].$

**Example** **5.** *Put* M={f;   f:   [0, 1]→[0, 1]   are  Bore  measurable }, *and define in the class* M *the operation*  ⋅ *as the natural product of fuzzy sets. It is easy to see that* M *is a product MV-algebra. Further, we define a state* s:  M→[0,1] *by the equality* $s(f)=\int_{0}^{1}f(x)dx,$ *for every* f∈M. *Let us consider the pairs* $\alpha =(a_{1},a_{2}),$ $\beta =(b_{1},b_{2}),$ *where* $a_{1}(x)=x,$ $a_{2}(x)=1-x,$ $b_{1}(x)=x^{2},\;b_{2}(x)=\;1-x^{2},$ x∈[0,1]. *It is obvious that* α *and* β *are partitions of* M. *Elementary calculations will show that they have the s-state values* $\frac{1}{2},\frac{1}{2}$ *and* $\frac{1}{3},\frac{2}{3}$ *of the corresponding elements, respectively, and the logical entropies* $h_{s}^{l}(\alpha )=\frac{1}{2},$ $h_{s}^{l}(\beta )=\frac{4}{9}.$ *The partition* α∨β= $\left(a_{1}\cdot b_{1},\;a_{1}\cdot b_{2},\;a_{2}\cdot b_{1},\;a_{2}\cdot b_{2}\right)$ *has the s-state values* $\frac{1}{4},\frac{1}{4},\frac{1}{12},\frac{5}{12}$ *of the corresponding elements, and the logical entropy:*

$$h_{s}^{l}(\alpha \vee \beta )=1-\left[{\left(\frac{1}{4}\right)}^{2}+{\left(\frac{1}{12}\right)}^{2}+{\left(\frac{1}{4}\right)}^{2}+{\left(\frac{5}{12}\right)}^{2}\right]≐0.694444\;.$$

*Simple calculations will show that* $h_{s}^{l}(\alpha /\beta )≐0.555556-0.305556\;=0.25\;.$ *By Equation (11) we obtain the logical mutual information of partitions* α *and* β:

$$I_{s}^{l}(\alpha ,\beta )=0.5-0.25=0.25.$$

*One can verify that:*

$$I_{s}^{l}(\alpha ,\beta )=h_{s}^{l}(\alpha )+h_{s}^{l}(\beta )-h_{s}^{l}(\alpha \vee \beta ).$$

**Remark** **7.** Let us remind that the product MV-algebra presented in the previous example represents an important class of fuzzy sets; it is called a full tribe of fuzzy sets (cf. [21,24,25]).

**Theorem** **7.** *If partitions* α *and* β *of* (M,⋅ ) *are statistically independent, i.e.,* s(a⋅b)=s(a)⋅s(b), *for every* a∈α, b∈β, *then:*

$$I_{s}^{l}(\alpha ,\beta )=h_{s}^{l}(\alpha )\cdot h_{s}^{l}(\beta ).$$

**Proof.**  Let $\alpha =(a_{1},\ldots ,a_{k}),$ $\beta =(b_{1},\ldots ,b_{l}).$ Using Equations (12) and (4) we obtain:

$$I_{s}^{l}(\alpha ,\beta )=1-\sum_{i=1}^{k}{\left(s(a_{i})\right)}^{2}+1-\sum_{j=1}^{l}{\left(s(b_{j})\right)}^{2}-1+\sum_{i=1}^{k}\sum_{j=1}^{l}{\left(s(a_{i})\cdot s(b_{j})\right)}^{2}\;=\left[1-\sum_{i=1}^{k}{\left(s(a_{i})\right)}^{2}\right]\cdot \left[1-\sum_{j=1}^{l}{\left(s(b_{j})\right)}^{2}\right]=h_{s}^{l}(\alpha )\cdot h_{s}^{l}(\beta ).$$

 ☐

As it is known, one of the most significant properties of Shannon entropy is additivity: if partitions A,B are statistically independent, then $h^{S}(A\vee B)=h^{S}(A)+h^{S}(B).$ Here, A∨B= {A∩B;  A∈A,B∈B}. In the case of logical entropy, the following property applies.

**Theorem** **8.** *If partitions* α *and* β *of* (M,⋅ ) *are statistically independent, then:*

$$1-h_{s}^{l}(\alpha \vee \beta )=(1-h_{s}^{l}(\alpha ))\cdot (1-h_{s}^{l}(\beta )).$$

**Proof.**  As a consequence of Theorem 7 and Equation (12), we obtain:

$$\left(1-h_{s}^{l}(\alpha ))\cdot (1-h_{s}^{l}(\beta ))=1-h_{s}^{l}(\alpha )-h_{s}^{l}(\beta )+h_{s}^{l}(\alpha )\cdot h_{s}^{l}(\beta )=1-h_{s}^{l}(\alpha )-h_{s}^{l}(\beta )+I_{s}^{l}(\alpha ,\beta \right)\;=1-h_{s}^{l}(\alpha )-h_{s}^{l}(\beta )+h_{s}^{l}(\alpha )+h_{s}^{l}(\beta )-h_{s}^{l}(\alpha \vee \beta )=1-h_{s}^{l}(\alpha \vee \beta ).$$

 ☐

In the following two theorems, using the concept of logical conditional mutual information, chain rules for logical mutual information in product MV-algebras are established.

**Definition** **8.** *Let* α,β,γ *be partitions of* (M,⋅ ). *The logical conditional mutual information of* α *and* β *assuming a realization of* γ *is defined by:*

(13) $$I_{s}^{l}(\alpha ,\beta /\gamma )=h_{s}^{l}(\alpha /\gamma )-h_{s}^{l}(\alpha /\beta \vee \gamma ).$$

**Remark** **8.** *It is easy to show that:*

$$I_{s}^{l}(\alpha ,\beta /\gamma )=I_{s}^{l}(\beta ,\alpha /\gamma ).$$

**Theorem** **9.** *For arbitrary partitions* α,β,γ *of* (M,⋅ ), *it holds:*

$$I_{s}^{l}(\alpha \vee \beta ,\gamma )=I_{s}^{l}(\alpha ,\gamma )+I_{s}^{l}(\beta ,\gamma /\alpha ).$$

**Proof.**  Elementary calculations will show that:

$$I_{s}^{l}(\alpha ,\gamma )+I_{s}^{l}(\beta ,\gamma /\alpha )=h_{s}^{l}(\gamma )-h_{s}^{l}(\gamma /\alpha )+h_{s}^{l}(\gamma /\alpha )-h_{s}^{l}(\gamma /\alpha \vee \beta )=I_{s}^{l}(\alpha \vee \beta ,\gamma ).$$

 ☐

**Theorem** **10.** *Let* $\alpha_{1},\alpha_{2},\ldots ,\alpha_{n},\gamma$ *be partitions of* (M,⋅ ). *Then:*

$$I_{s}^{l}(\vee_{i=1}^{n}\alpha_{i},\gamma )=I_{s}^{l}(\alpha_{1},\gamma )+\sum_{i=2}^{n}I_{s}^{l}(\alpha_{i},\gamma /\alpha_{1}\vee \ldots \vee \alpha_{i-1}).$$

**Proof.**  It follows by applying Equations (11), (8), (10), and (13). ☐

**Definition** **9.** *Let* α,β,γ *be partitions of* (M,⋅ ). *We say that* α *and* γ *are conditionally independent given* β *if* $I_{s}^{l}(\alpha ,\gamma /\beta )=h_{s}^{l}(\alpha /\beta )\cdot h_{s}^{l}(\gamma /\beta ).$

**Theorem** **11.** *Let* α,β,γ *be partitions of* (M,⋅ ). *If* α *and* γ *are conditionally independent given* β, *then:*

$$I_{s}^{l}(\alpha \vee \beta ,\gamma )=I_{s}^{l}(\beta ,\gamma )+h_{s}^{l}(\alpha /\beta )\cdot h_{s}^{l}(\gamma /\beta ).$$

**Proof.**  Using Theorem 9 we get:

$$I_{s}^{l}(\alpha \vee \beta ,\gamma )=I_{s}^{l}(\beta ,\gamma )+I_{s}^{l}(\alpha ,\gamma /\beta )=I_{s}^{l}(\beta ,\gamma )+h_{s}^{l}(\alpha /\beta )\cdot h_{s}^{l}(\gamma /\beta ).$$

 ☐

## 5. Logical Cross Entropy and Logical Divergence in Product MV-Algebras

In this section, we define the notions of logical cross entropy and logical divergence in product MV-algebras. The proposed notions are analogies of the concepts of logical cross entropy and logical divergence introduced by Ellerman in [50]. For illustration, we provide some numerical examples.

**Definition** **10.** Let $\alpha =(a_{1},\ldots ,a_{n})$ be a partition in a product MV-algebra (M,⋅ ), and s,t∈S(M). We define the logical cross entropy of states s,t with respect to α by the formula:

$$h_{\alpha }^{l}(s\|t)=\sum_{i=1}^{n}s(a_{i})\;(1-t(a_{i})).$$

**Remark** **9.** *Since* $\sum_{i=1}^{n}s(a_{i})\;=1,$ *we can also write:*

$$h_{\alpha }^{l}(s\|t)=1-\sum_{i=1}^{n}s(a_{i})t(a_{i}).$$

*Evidently, the logical cross entropy* $h_{\alpha }^{l}(s$ *‖* t) *is symmetric and it is always nonnegative. If states* s,   t *are identical over* α *(i.e.,* $s(a_{i})=t(a_{i}),$ *for* i=1,2,…,n *), then* $h_{\alpha }^{l}(s$ *‖* $t)=h_{s}^{l}(\alpha ).$

**Definition** **11.** Let $\alpha =(a_{1},\ldots ,a_{n})$ be a partition in a product MV-algebra (M,⋅ ), and s,t∈S(M). We define the logical divergence of states s,t with respect to α by the formula:

$$d_{\alpha }^{l}(s\|t)=\frac{1}{2}\sum_{i=1}^{n}{\left(s(a_{i})-t(a_{i})\right)}^{2}\;.$$

**Remark** **10.** *It is evident that* $d_{\alpha }^{l}(s$ *‖* $t)=d_{\alpha }^{l}(t$ *‖* s), *and* $d_{\alpha }^{l}(s$ *‖* t)≥0 *with the equality if and only if the states* s,   t *are identical over* α. *As in the case of K-L divergence, the logical divergence is not a distance metric because it does not satisfy the triangle inequality (as shown in the example that follows). Notice that its square root (with or without the* $\frac{1}{2}$ *factor) is a natural distance metric.*

**Example** **6.** *Consider any product MV-algebra* (M,⋅ ) *and states* $s_{1},s_{2},s_{3}$ *defined on it. Let* a∈M *with* $s_{1}(a)=p_{1},$ $s_{2}(a)=p_{2},$ $s_{3}(a)=p_{3},$ *where* $p_{1},p_{2},p_{3}\in (0,1).$ *Then* $s_{1}(u-a)=1-p_{1},$ $s_{2}(u-a)$ $=1-p_{2},$ *and* $s_{3}(u-a)=1-p_{3}.\;$ *Put* $p_{1}=\frac{1}{2},$ $p_{2}=\frac{1}{3},$ $p_{3}=\frac{1}{4},$ *and consider the partition* α=(a,u−a) *of* (M,⋅). *Let us calculate:*

$$d_{\alpha }^{l}(s_{1}\|s_{2})=\frac{1}{2}{\left(s_{1}(a)-s_{2}(a)\right)}^{2}\;+\frac{1}{2}{\left(s_{1}(u-a)-s_{2}(u-a)\right)}^{2}={\left(p_{1}-p_{2}\right)}^{2}=\frac{1}{36}.$$

*Analogously:*

$$d_{\alpha }^{l}(s_{1}\|s_{3})={\left(p_{1}-p_{3}\right)}^{2}=\frac{1}{16},\;and\;d_{\alpha }^{l}(s_{2}\|s_{3})={\left(p_{2}-p_{3}\right)}^{2}=\frac{1}{144}.$$

*Evidently,*

$$d_{\alpha }^{l}(s_{1}\|s_{3})>d_{\alpha }^{l}(s_{1}\|s_{2})+\;d_{\alpha }^{l}(s_{2}\|s_{3}).$$

*The result means that the triangle inequality of the logical divergence in product MV-algebras does not apply, in general.*

**Theorem** **12.** *Let* α *be a partition of a product MV-algebra* (M,⋅ ). *Then, for every states* s,t *defined on* (M,⋅ ), *it holds:*

$$d_{\alpha }^{l}(s\|t)=h_{\alpha }^{l}(s\|t)-\frac{1}{2}(h_{s}^{l}(\alpha )+h_{t}^{l}(\alpha )).$$

**Proof.**  Assume that $\alpha =(a_{1},\ldots ,a_{n}).$ Let us calculate:

$$h_{\alpha }^{l}(s\|t)-\frac{1}{2}(h_{s}^{l}(\alpha )+h_{t}^{l}(\alpha ))=1-\sum_{i=1}^{n}s(a_{i})t(a_{i})-\frac{1}{2}\left(1-\sum_{i=1}^{n}{\left(s(a_{i})\right)}^{2}\right)-\frac{1}{2}\left(1-\sum_{i=1}^{n}{\left(t(a_{i})\right)}^{2}\right)=\frac{1}{2}\sum_{i=1}^{n}{\left(s(a_{i})-t(a_{i})\right)}^{2}\;=\;d_{\alpha }^{l}(s\;\|t).$$

 ☐

**Remark** **11.** *As a simple consequence of the previous theorem and the logical information inequality* $d_{\alpha }^{l}(s$ *‖* t)≥0 *(with the equality if and only if the states* s,   t *are identical over* α) *we get that* $h_{\alpha }^{l}(s$ *‖* t)≥ $\frac{1}{2}(h_{s}^{l}(\alpha )+h_{t}^{l}(\alpha ))$ *with the equality if and only if the states* s,   t *are identical over* α.

**Example** **7.** *Consider the product MV-algebra* (M,⋅ ) *from Example 4 and the real functions* $F_{1},\;F_{2}$ *defined by* $F_{1}(x)=x,\;F_{2}(x)=x^{2},$ *for every* x∈[0,1]. *We define on the product MV-algebra* (M,⋅ ) *two states* $s_{1},\;s_{2}$ *by the formulas:*

$$s_{1}(I_{A})=\int_{0}^{1}I_{A}(x)dF_{1}(x)=\int_{0}^{1}I_{A}(x)dx,$$

$$s_{2}(I_{A})=\int_{0}^{1}I_{A}(x)dF_{2}(x)=\int_{0}^{1}I_{A}(x)2xdx,$$

*for any element* $I_{A}$ *of* M. *The partition* $\alpha =\left(I_{\left[0,\;\frac{1}{2}\right]},\;I_{\left[\frac{1}{2},\;1\right]}\right)$ *has the* $s_{1}$-*state values* $\frac{1}{2},\frac{1}{2}$ *of the corresponding elements, and the* $s_{2}$-*state values* $\frac{1}{4},\frac{3}{4}$ *of the corresponding elements. Elementary calculations will show that* $h_{s_{1}}^{l}(\alpha )=\frac{1}{2},$ *and* $h_{s_{2}}^{l}(\alpha )=\frac{3}{8}$. *Further we get:*

$$h_{\alpha }^{l}(s_{1}\|s_{2})=\sum_{i=1}^{2}s_{1}(a_{i})\;(1-s_{2}(a_{i}))=\frac{1}{2},\;and\;d_{\alpha }^{l}(s_{1}\|s_{2})=\frac{1}{2}\sum_{i=1}^{2}{\left(s_{1}(a_{i})-s_{2}(a_{i})\right)}^{2}=\frac{1}{16}.$$

*It is now possible to verify that:*

$$d_{\alpha }^{l}(s_{1}\|s_{2})=h_{\alpha }^{l}(s_{1}\|s_{2})-\frac{1}{2}(h_{s_{1}}^{l}(\alpha )+h_{s_{2}}^{l}(\alpha )),$$

*and*

$$h_{\alpha }^{l}(s_{1}\|s_{2})\geq \frac{1}{2}(h_{s_{1}}^{l}(\alpha )+h_{s_{2}}^{l}(\alpha )).$$

## 6. Conclusions

In [48], the authors introduced the concepts of mutual information and K-L divergence in product MV-algebras and derived the fundamental properties of these quantities. Naturally, the presented theory is based on the Shannon entropy function (Equation (1)). The aim of this paper was to construct a relevant theory on product MV-algebras for the case when the Shannon entropy function is replaced by the logical entropy function (Equation (2)). The main results of the paper are contained in Section 3, Section 4 and Section 5.

In Section 3, we have proposed the concepts of logical entropy and logical conditional entropy of partitions in product MV-algebras and examined their properties. Among others, the concavity of logical entropy has been proved. In Section 4, the notions of logical entropy and logical conditional entropy have been exploited to define the logical mutual information for the examined case of product MV-algebras. We have shown basic properties of these quantities. Moreover, chain rules for logical entropy and logical mutual information for the studied case of product MV-algebras were derived. In the final section, the notions of logical cross entropy and logical divergence in product MV-algebras were proposed. To illustrate the developed theory, several numerical examples are included in the paper.

As already mentioned in Section 4 (see Example 5), an important case of product MV-algebras is the full tribe M of fuzzy sets. We note that in [21] (see also [24,25]) the entropy of Shannon type on the full tribe M of fuzzy sets was examined. In a natural way, all results, based on the logical entropy function (2), provided by the theory developed in the paper may be applied also to the case of a full tribe of fuzzy sets. 
